# Computational modeling of bone fracture non-unions: four clinically relevant case studies

**DOI:** 10.1186/s40482-015-0004-x

**Published:** 2015-12-18

**Authors:** Aurélie Carlier, Johan Lammens, Hans Van Oosterwyck, Liesbet Geris

**Affiliations:** Biomechanics Section, KU Leuven, Celestijnenlaan 300 C, PB 2419, 3000 Leuven, Belgium; Prometheus, Division of Skeletal Tissue Engineering, KU Leuven, O&N 1, Herestraat 49, PB 813, 3000 Leuven, Belgium; Department of Orthopaedics, University Hospitals of KU Leuven, KU Leuven, Weligerveld 1-blok 1, 3212 Pellenberg, Belgium; Biomechanics Research Unit, Department of Aerospace and Mechanical Engineering, University of Liege, Chemin des Chevreuils 1-BAT 52/3, 4000 Liege 1, Belgium

**Keywords:** Computational model, Fracture healing, Non-union

## Abstract

The human skeleton has a remarkable regeneration capacity. Nevertheless, 5–10 % of the bone fractures fails to heal and develops into a non-union which is a challenging orthopedic complication requiring complex and expensive treatment. This review paper will discuss four different computational models, each capturing a particular clinical case of non-union: non-union induced by reaming of the marrow canal and periosteal stripping, non-union due to a large interfragmentary gap, non-union due to a genetic disorder [i.e. NF1 related congenital pseudoarthrosis of the tibia (CPT)] and non-union due to mechanical overload. Together, the four computational models are able to capture the etiology of a wide range of fracture non-union types and design novel treatment strategies thereof. Further research is required to corroborate the computational models in both animal and human settings and translate them from bench to bed side.

## Background

In case of injury, the majority of bone fractures can heal without the production of scar tissue. Unfortunately, fracture healing complications, such as delayed and non-unions, are associated with 5–10 % of the over 6 million fractures occurring annually in the USA [1, 2]. Fracture non-unions are challenging orthopedic complications requiring complex and expensive treatment including multiple surgical procedures and prolonged hospital stay [3–5]. As such, the resulting socio-economic burden is significant and rising according to the 2010 Global Burden of Disease study where musculoskeletal disorders accounted for 6–8 % of total disability-adjusted life years (DALYs) [6].

Although the field of orthopedics has experienced great advancements in the last decades, more knowledge of the complex physiological process of bone healing is a prerequisite for the prevention and effective treatment of complex fractures. (Patient-specific) Computational models have the potential to cope with this complexity. Moreover, computational models can help to integrate all the relevant, patient-specific information into a personalized diagnosis and optimal treatment.

This article will focus on the use of in silico models to improve our fundamental understanding of impaired bone regeneration and to design novel treatment strategies. It will first briefly summarize the biology of bone regeneration, including possible complications and treatment options. Subsequently, the added value of computational models will be illustrated with four different clinical cases of non-unions: non-union induced by reaming of the marrow canal and periosteal stripping, non-union due to a large interfragmentary gap, non-union due to a genetic disorder (i.e. NF1 related congenital pseudoarthrosis of the tibia (CPT)) and non-union due to mechanical overload. Finally, some prospects and conclusions will be formulated.

## Biology of bone fracture healing

### Normal and impaired bone regeneration

**Fig. 1:**
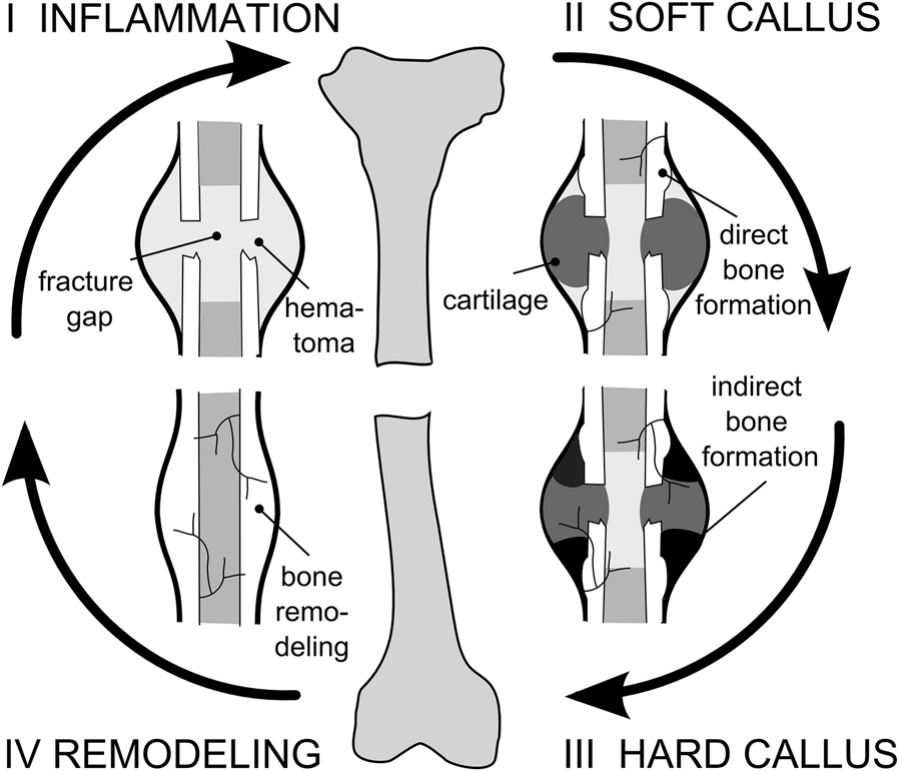
Schematic representation of the four different phases of fracture healing. *I* the inflammatory phase, *II* the soft tissue callus phase, *III* the hard callus phase and *IV* the remodeling phase

Primary bone healing, during which the fracture will heal similar to the normal bone remodeling process, will only occur under optimal mechanical conditions, i.e. a mechanically stabilized fracture with either extremely low interfragmentary movement or bony fragments that are under compression. The more common type of healing, i.e. secondary bone healing, is associated with a low degree of stability and the formation of a periosteal callus. Briefly, the characteristic course of long bone healing can be subdivided in four main stages (Fig. 1): (1) the “inflammation phase” where the trauma site becomes hypoxic and is invaded by inflammatory cells, fibroblasts and mesenchymal stem cells, (2) the “reparative phase” which starts with the production of a cartilaginous and fibrous tissue template (“soft callus phase”), later invaded by new blood vessels and replaced by a bony callus through endochondral ossification (“hard callus phase”), (3) the final “remodeling phase” during which the woven bone is replaced by lamellar bone and the vasculature is reorganized.

Although bone has a unique restorative capacity, the conditions for spontaneous bone healing are not always present leading to a delayed union or a non-union. The former is generally defined as the eventual bony union after an atypical long period of healing, in comparison to the normal healing period [7, 8]. The latter is characterized by an absence of healing during at least 6–12 months (in humans). The different types of fracture non-unions, i.e. hypertrophic, atrophic or oligotrophic, and synovial pseudarthrosis are classified based on their radiographic and histological appearance [7, 9].

Hypertrophic non-unions are mainly defined by an abnormal vascularity and abundant callus formation. In this type of non-union, the excessive motion at the fracture site prevents the bony bridging although the essential biological factors are present [7]. Atrophic non-unions, however, are the result of inadequate biological conditions. They show little callus formation around the gap, which is mostly filled with fibrous tissue and little or no mineral deposition as evidenced by the blunted bony ends on radiographs [7]. Oligotrophic non-unions combine the radiographic characteristics of atrophic non-unions, i.e. little to no callus formation, with the biological characteristics of hypertrophic non-unions, i.e. normal biological activity [10]. In a synovial pseudarthrosis the fracture gap of the non-union is a fluid-filled cavity with a synovial-like membrane.

### Current treatment strategies

Of the classical therapeutic methods currently available to treat non-unions, amputation is the treatment of last resort [11]. Even when other treatment options are available, the choice is complex since at a more phenomenological level the outcome of bone healing depends on many mechanical and biological risk factors, such as excess motion, the interfragmentary gap size [12], the type of fracture [12–14], the particular bone [11], location of the trauma within the bone [11], loss of blood supply [15]. Additional injuries such as severe periosteal and soft-tissue trauma [13, 14] as well as other preexisting patients risk factors including old age [16], cachexia and malnutrition [17], immune compromise [18], genetic disorders (e.g. neurofibromatosis type 1 [19]), osteoporosis [20], anticoagulants [21], smoking [22] and anti-inflammatory agents [23], may also compromise the optimal treatment.

Strategies for hypertrophic non-unions traditionally aim to restore the optimal mechanical environment for fracture healing [7, 24]. Enhanced stabilization and progressive compression such as in the Ilizarov method can allow to convert the abnormal fibrous, cartilaginous and adipose tissues between the mobile bony ends into bone without the need to remove them. In case of plate osteosynthesis a debridement is usually performed as the fracture is exposed anyway, whereas in intramedullary nailing the reaming of the canal contributes to the refreshment of the fracture zone. The key to success either with plates, intramedullary nails or external fixators is the reduction of any excessive motion allowing a sound biomechanical condition for bone healing [7, 24–28].

Successful healing of the more challenging (atrophic) non-unions will, however, not only require the removal of scar tissue and adequate stabilization of the fracture but also biological support (e.g. bone grafting, administration of growth factors) [7]. Large quantities of bone are not only required for compromised bone healing processes, they are also indispensable for skeletal reconstructions of large bone defects created by trauma, infection, tumor resection and skeletal abnormalities [29]. A widely used approach to stimulate or augment bone formation is distraction osteogenesis, a clinical procedure where bone regeneration is induced between two gradually distracted bony surfaces [30, 31]. This principle is applied in the bone transport technique, allowing huge defects to be replaced [32, 33]. However, due to the length of the treatment, as well as the technical demands and complications associated with distraction osteogenesis [30, 31], a number of bone grafting methods are more commonly performed in clinical practice when the defect size is not too extensive. Autologous bone grafting, i.e. the process by which bone is harvested from one anatomical site and transplanted to another site in the same patient, is still considered the “gold standard” since it effectively combines the required osteoinductive, osteogenic and osteoconductive properties [34]. It has, however, several limitations which include donor site pain, increased blood loss, increased surgery times, increased risk for donor site infection and limited supply [35]. An even more complex autologous bone transplant is the vascularized bone graft such as the fibula, which can be used to replace long-sized bone defects [36]. Allogeneic bone grafts, where bone is harvested from human cadavers, sterilized and transplanted to the patient, lack donor site morbidity but are expensive and have an increased risk of viral transmission [7]. Moreover, they have very limited biological activity as they are only osteoconductive without any osteogenic capacity and only sporadically a small osteoinductive capacity. As such they are inappropriate to treat atrophic non-unions. The use of synthetic calcium salt-based bone substitutes is sometimes suggested as an alternative to both autologous and allogeneic grafts since they are inexpensive and lack the risks of donor site morbidity and viral transmission [37]. They are, however, only osteoconductive which limits their potential biological role in fracture healing [7, 38] and as such they cannot be recommended as a stand-alone treatment in hampered bone healing. The use of a “Masquelet-membrane” is to be considered as an enhanced bone grafting method which consists of two steps. In the first stage a polymethylmetacrylate cement spacer is placed in the defect which induces the formation of a vascularized membrane. In the second stage a non-vascularized graft will be inserted in the newly formed vascularized envelope which serves as a source of oxygen, nutrients and a cocktail of important growth factors [39, 40]. Besides bone grafting also bioactive molecules have been used to augment fracture healing. BMP-2 and BMP-7 have been shown to have significant osteogenic and angiogenic properties, which has led to their use in a variety of clinical conditions including non-unions, open fractures and joint fusions [29, 41]. An alternative approach is the local application of platelet-rich plasma, which is rich in many of the growth factors implicated in bone regeneration [42]. Another promising strategy, which could potentially overcome the limitations of current bone regeneration therapies, is tissue engineering where an optimal bone healing microenvironment is created by seeding cells (osteogenesis) and growth factors (osteoinduction) on biocompatible scaffolds (osteoconduction) that will be implanted in a mechanically stabilized defect [43].

## In silico modeling of bone fracture non-unions

**Fig. 2:**
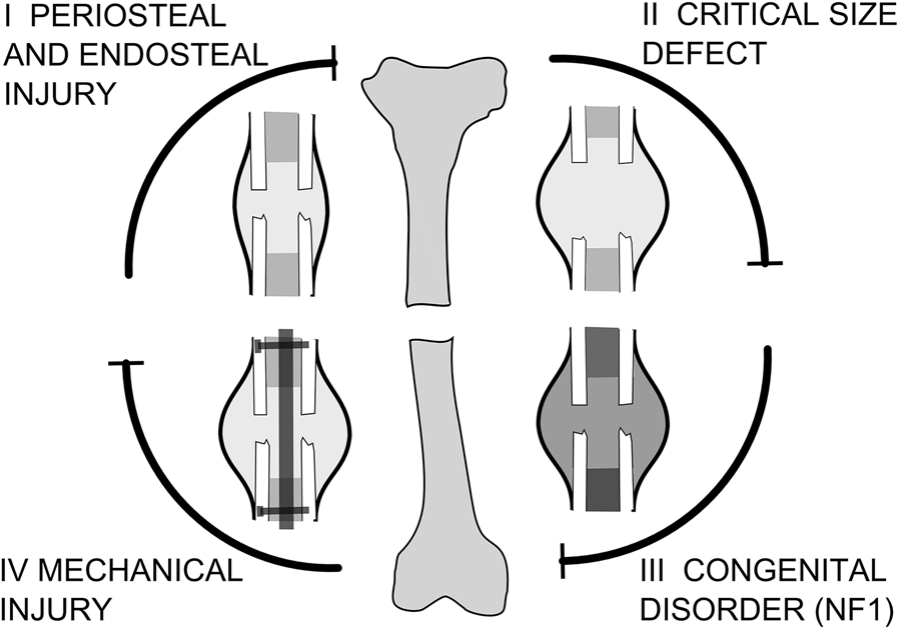
Schematic representation of the four different causes of non-union. *I* non-union induced by reaming of the marrow canal and periosteal stripping, *II* non-union due to a critical size defect, *III* non-union due to a genetic disorder, i.e. NF1 related congenital pseudarthrosis of the tibia and *IV* non-union due to mechanical overload

Over the last decades computational models of fracture healing have progressed from static, linear elastic models to dynamic poroelastic analyses, accounting for callus growth and several biological factors including growth factors, cells and vascularization [44]. In this section we will use four different clinically relevant case-studies to illustrate the potential of computational models, initially developed for normal fracture healing, to investigate the etiology and treatment of fracture non-unions (Fig. 2). For further information on the bioregulatory and mechanoregulatory algorithms used in these computational models we refer the reader to some reviews [44–47].

### Modeling framework

**Fig. 3:**
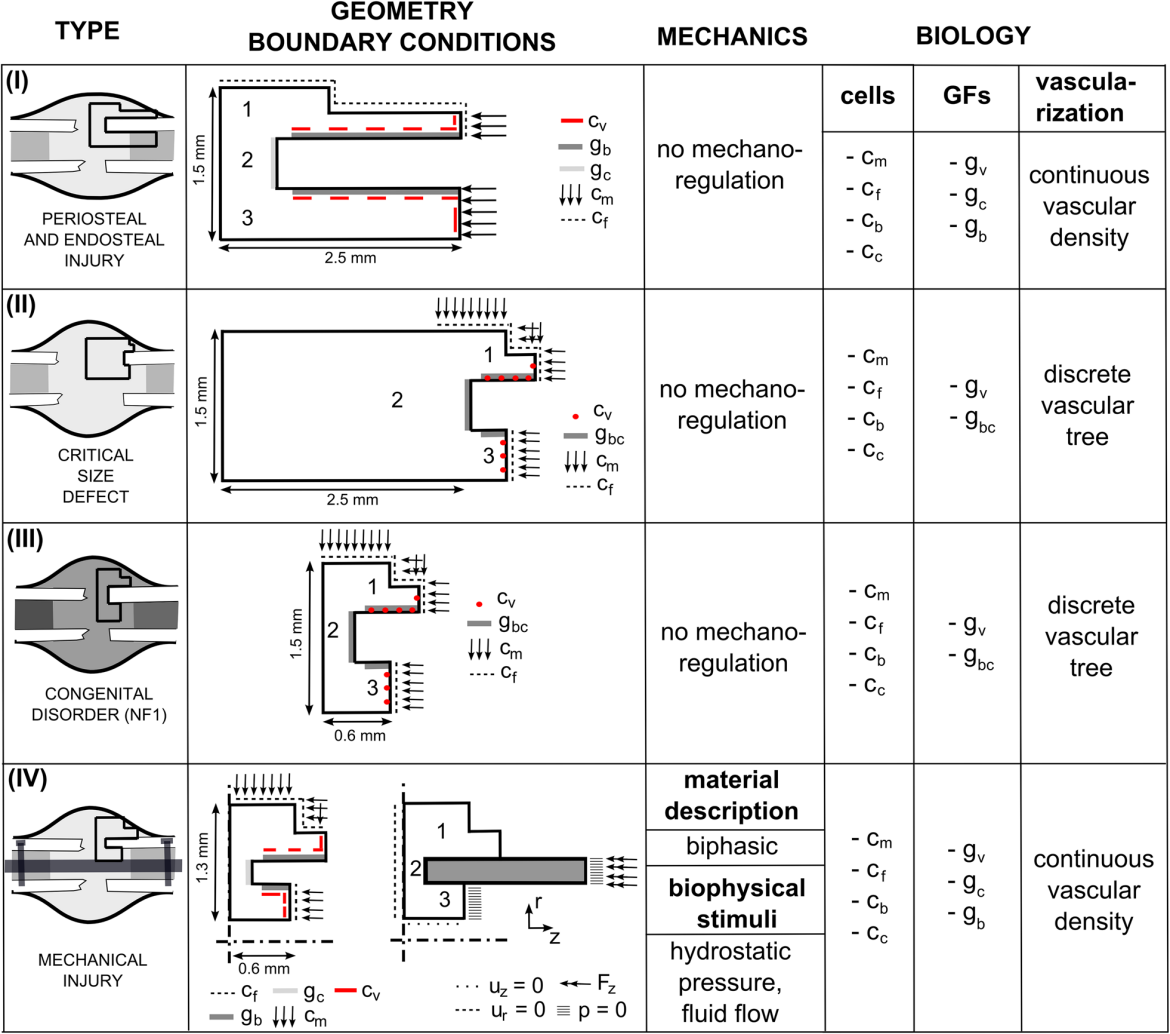
Comparison of the computational frameworks used to model the four different causes of non-union. *I* non-union induced by reaming of the marrow canal and periosteal stripping [48], *II* non-union due to a critical size defect [49], *III* non-union due to a genetic disorder, i.e. NF1 related congenital pseudarthrosis of the tibia [50] and *IV* non-union due to mechanical overload [51]. *GFs* growth factors, *c*_*m*_ mesenchymal stem cell density, *c*_*f*_ fibroblast cell density, *c*_*b*_ osteoblast cell density, *c*_*c*_ chondrocyte cell density, *g*_*bc*_ generic osteochondrogenic growth factor, *g*_*b*_ generic osteogenic growth factor, *g*_*c*_ generic chondrogenic growth factor, *g*_*v*_ generic angiogenic growth factor, *c*_*v*_ endothelial cell (density), *u*_*z*_ displacement in the z-direction, *u*_*r*_ displacement in the r-direction, *p* hydrostatic pressure, *F*_*z*_ applied loading, *1* periosteal callus, *2* intercortical callus, *3* endosteal callus

The four clinically-relevant case-studies presented here are all derived from the same general modeling framework. More specifically, the general mathematical model describes the key processes of bone regeneration as a function of time and space, using a number of continuum-type of variables (such as growth factor concentration, cell densities and matrix densities). The bone regeneration processes are captured by a system of partial differential equations (PDEs) of the taxis-diffusion–reaction type of which the general structure is as follows:

1 $$\frac{{\partial \vec{c}_{m} }}{\partial t} = \nabla .\left[ {D_{cm} \left( {\vec{c}_{m} } \right)\nabla \vec{c}_{m} - \vec{c}_{m} \mathop \sum \limits_{i = 1}^{n} f_{i} (\vec{c})\nabla \vec{c}_{i} } \right] + f_{0} \left( {\vec{c}_{m} ,\vec{c}} \right)$$

2 $$\frac{{\partial \vec{c}}}{\partial t} = D\varDelta \vec{c} + \vec{g}\left( {\vec{c}_{m} ,\vec{c}} \right)$$

where *t* represents time, $\vec{x}$ the space and $\vec{c}_{m} (t,\vec{x})$ the density of a migrating cell type (i.e. mesenchymal stem cell, fibroblast and endothelial cells). $\vec{c}(t,\vec{x})$ represents the vector of the other cell densities, matrix densities and growth factor concentrations. $D_{cm} \left( {\vec{c}_{m} } \right)$ and *D* are the diffusion coefficients, $f_{i} (\vec{c})$ represents the taxis coefficients for chemotaxis and haptotaxis. $f_{0} \left( {\vec{c}_{m} ,\vec{c}} \right)$ and $\vec{g}\left( {\vec{c}_{m} ,\vec{c}} \right)$ are the reaction terms describing cell proliferation, differentiation and apoptosis as well as matrix and growth factor production and decay. The equations are solved on a simplified (fixed) geometrical domain of the fracture callus (Fig. 3). The current implementation of the framework assumes a constant callus size and axisymmetry so that only a quarter of the domain is simulated (Fig. 3). In order to ensure the existence, uniqueness and non-negativity of the solution, the system of Eqs. (1)–(2) is complemented by suitable initial and boundary conditions (Fig. 3), which are dependent on the specific case-study.

Although the four cases-studies are based on the same general framework, there are some important differences which are detailed below and summarized in Fig. 3. Firstly, only case-study IV includes the role of mechanical influences on the healing processes for which the finite element analyses were carried out in MSC.Marc Mentat (Version 2005r2, Palo Alto, USA). The bioregulatory model of all case studies is solved in a custom finite volume code using Matlab (The MathWorks, Inc.). Secondly, case studies I and IV use a continuous description of bone regeneration (tissue level), including a continuous vascular density, whereas case studies II and III use a multi-scale description that combines a continuous tissue-level with a discrete cellular level (i.e. the vascular tree) and a detailed intracellular signaling network (i.e. Dll4-Notch signaling in the endothelial cells). Thirdly, case studies I and IV discriminate between a generic osteogenic (*g*_*b*_) and chondrogenic (*g*_*c*_) growth factor, whereas case studies II and III include one generic osteochondrogenic (*g*_*bc*_) growth factor, whose influence of differentiation is either chondrogenic or osteogenic depending on the local oxygen tension. A complete description of the set of equations, the boundary and initial conditions, the parameter values, implementation details as well as some underlying assumptions and simplifications can be found in previous publications of the respective cases: case I [48], case II [49], case III [50], case IV [51].

### Periosteal and endosteal injury

**Fig. 4:**
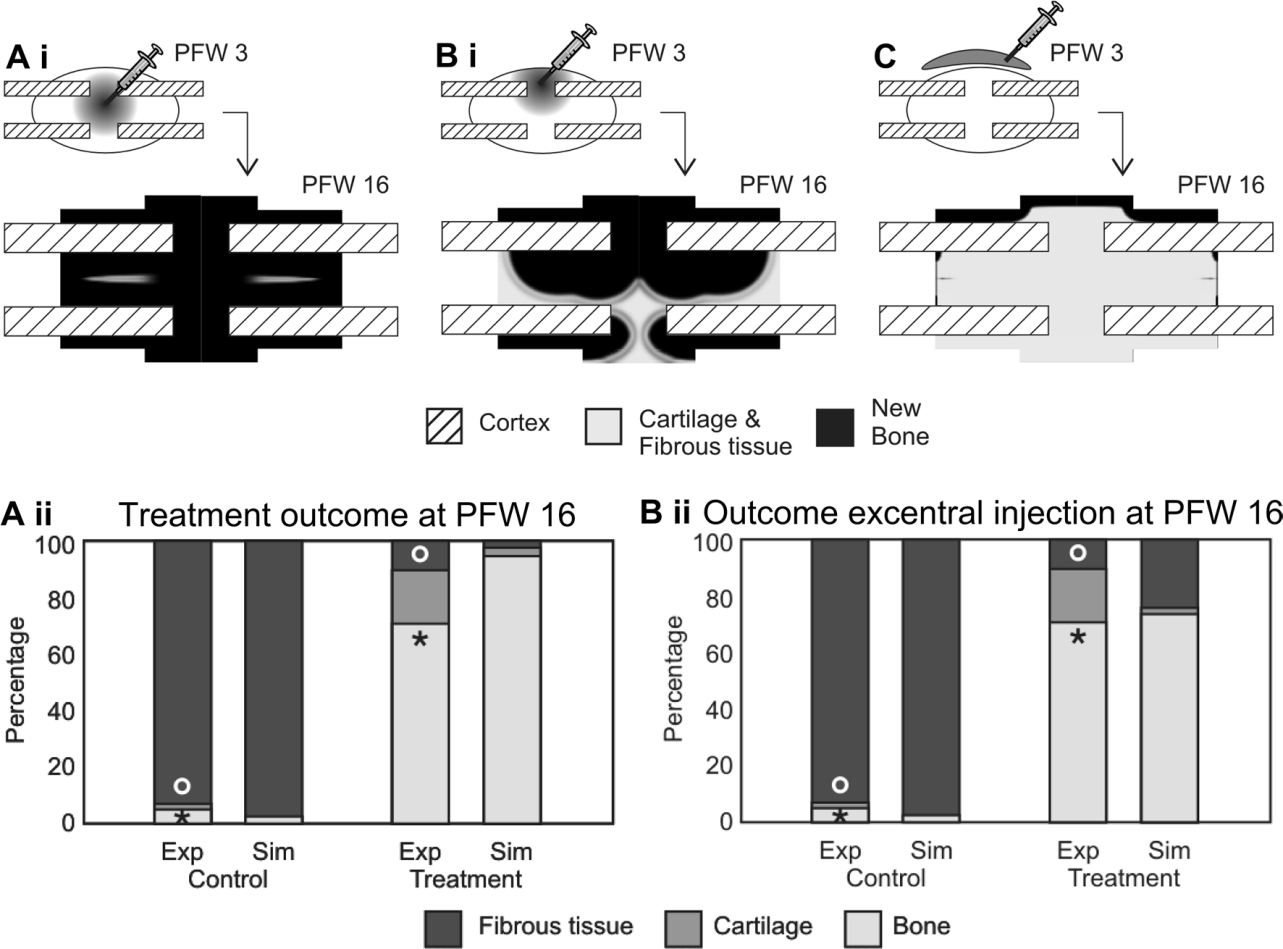
In silico and in vivo results of the effects of MSC transplantation on atrophic non-union formation. **Ai** In silico results for the treatment with the cell transplant injected in the center of the callus. **Aii** A comparison of experimentally measured (Exp) and numerically calculated (Sim) tissue constituents present within the interfragmentary gap of control (carrier solution injected) and treatment (MSC transplant) groups (^o^*p < 0.005, students *t* test). Simulation results are shown for a central injection of the carrier solution. **Bi** In silico results for the treatment with the cell transplant injected excentrally in the callus. **Bii** A comparison of experimentally measured (Exp) and numerically calculated (Sim) tissue constituents present within the interfragmentary gap of control (carrier solution injected) and treatment (MSC transplant) groups (^o^*p < 0.005, students t-test). Simulation results are shown for an excentral injection of the carrier solution. **C** Simulation results for the treatment with the cell transplant injected outside the callus. *PFW* post fracture week. (adapted from Geris et al. [48], licensed under CC BY 4.0)

As described above, atrophic non-unions are typically the result of inadequate biological conditions [7], e.g. a limited blood supply, a lack of growth factors and/or progenitor cells caused by periosteal and endosteal injury. These different aspects of atrophic non-unions, as well as some treatment strategies were rigorously investigated by Geris et al. in a combined experimental-modeling approach [48]. The experimental set-up consisted of a clinically relevant model of atrophic non-union in the rat. In short, a 1 mm gap was introduced at the site of the tibial osteotomy, the periosteum was stripped and the intramedullary canal curetted for a distance of one tibial diameter, both proximally and distally [52]. The fractures were fixated with a circular frame external fixator using nylon rings and copper screws [52]. In this study, the focus was on the bioregulatory aspects of atrophic non-unions so the influence of mechanical stimuli was neglected in the computational model. To simulate the atrophic non-union case, the domain was extended at the distal end (away from the fracture site) to represent the stripping of the periosteum and reaming of the marrow canal in the experimental set-up (Fig. 3I). Interestingly, only when both the periosteum was stripped and the marrow canal was reamed the occurrence of a non-union was predicted. In other cases, the removal of a MSC source resulted in a delayed healing, which confirmed the key role of progenitor cells in the beginning of the healing cascade. After careful validation of the mathematical model, a possible treatment strategy was designed in silico and tested in vivo in order to restore the adequate biological conditions lacking in atrophic non-unions. At postosteotomy week (POW) three, 1 ml of MSCs was administered in the center of the callus at a concentration of 10^6^ cells/ml (Fig. 4Ai). POW 3 was chosen to allow recovery of the blood supply in the gap since blood vessel formation is often delayed in (atrophic) non-union cases. The model of Geris et al. predicted a gradual increase in bone formation after administration of MSCs up till POW 16 (Fig. 4Ai) [48]. This was corroborated by the experimental results that showed bony bridging in three of the four treatment animals and significantly more bone formation in the treatment group than in the control group (Fig. 4Aii). Interestingly, the exact location of the injection appeared to be crucial, with excentral injection leading to unicortical bridging (Fig. 4Bi) or even the formation of a bony layer at the outside of the callus which would prevent the invasion of other cells from the surrounding tissues (Fig. 4C) [48].

### Non-union due to large interfragmentary gap

**Fig. 5:**
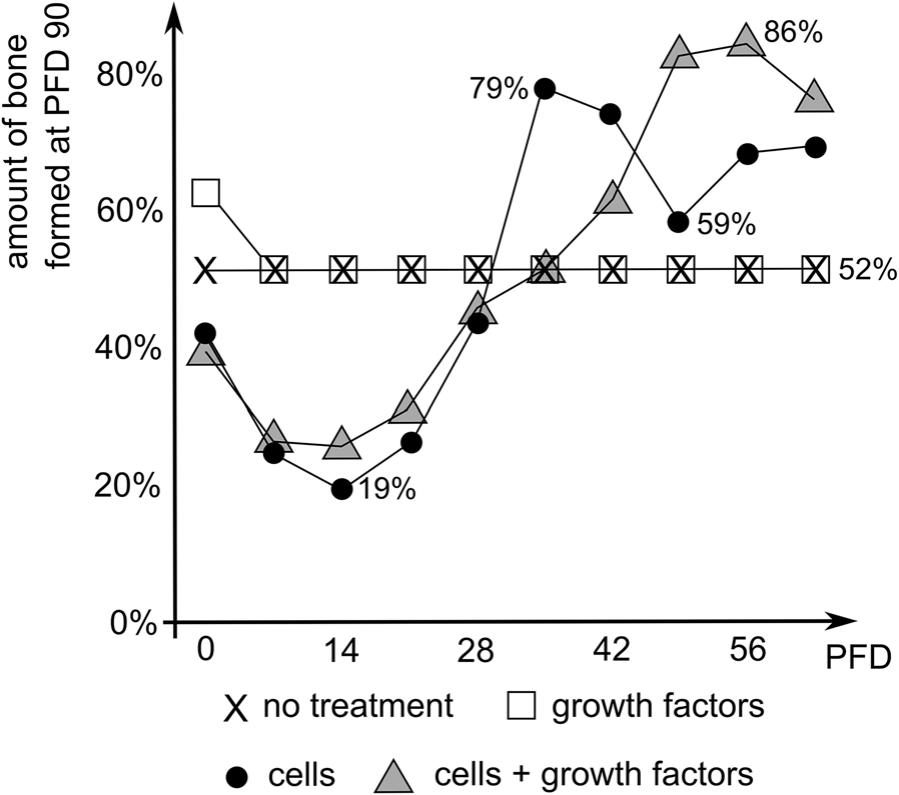
Predicted amount of bone formation at post fracture day (PFD) 90 in a critical size defect surrounded by a compromised environment as a function of the PFD at which a particular treatment was initiated. The compromised environment was modeled as the absence of any influx from the host environment and the treatment consisted of a single injection of growth factors, cells or a combination thereof. Note that a delayed injection of only growth factors does not improve the bone formation outcome. The injection of MSCs is only beneficial when delayed until PFD 35 whereas the combination yields better results at PFD 56. Importantly, although the amount of bone is increased, none of the treatments results in complete healing of the bone defect (adapted from [49], licensed under CC BY 4.0)

A large interfragmentary gap is a known biological risk factor for the development of a non-union [12]. In order to investigate the influence of the gap size on the healing outcome, Carlier et al. first established an in silico and in vivo murine non-union model [53]. They demonstrated that the in silico murine bone defect becomes critical at 3 mm, which corresponds to other experimental observations: 2 mm [54], 3 mm [55], 3.5 mm [56] and 4 mm [57]. They also showed that the biological potential of the fracture callus, i.e. the amount of stem cells and growth factors present in the fracture callus after the inflammatory phase, has an important impact on the final amount of bone formation. In critical size defects the biological potential is, however, not sufficient to result in complete healing due to insufficient vascularization of the central callus area, leading to hypoxic conditions and cell death [53]. In a next step Carlier et al. applied different combinations of boundary conditions to the in silico model to explore the role of the surrounding muscle envelope as a source for vascularization, progenitor cells and growth factors. They conclude that the host environment, and more specifically its role as a source for vascularization is critical for successful bone healing. Intrigued by these results, Carlier et al. [49] designed in silico treatment strategies for critical size defects surrounded by a permissive and a compromised host environment. In a permissive host environment, the fracture callus is partially supplied by blood vessels from the overlying muscle which improves the bone formation but nevertheless results in a non-union. A compromised environment is characterized in the in silico model by the absence of any influx from the host environment. Interestingly, the in silico model predicts that the injection of growth factors at day 0 results in a complete healing in a permissive host environment. The injection of cells or a combination product improve the bone healing outcome but do not rescue the healing process [53]. In a compromised environment, the injection of MSCs or a combination of MSCs and growth factors elicited an improved bone healing response (although without reaching full bridging) in silico if the environment is sufficiently vascularized to sustain the cell viability, which according to the model results meant a delay of injection until a certain time point (i.e. 35 days for MSCs, 49 days for the combination product) (Fig. 5) [53]. Growth factor injections at later time points in a compromised environment are, however, to no avail, since there are no cells left in the central callus area due to the hypoxic conditions (Fig. 5).

### Non-union due to a genetic disorder

**Fig. 6:**
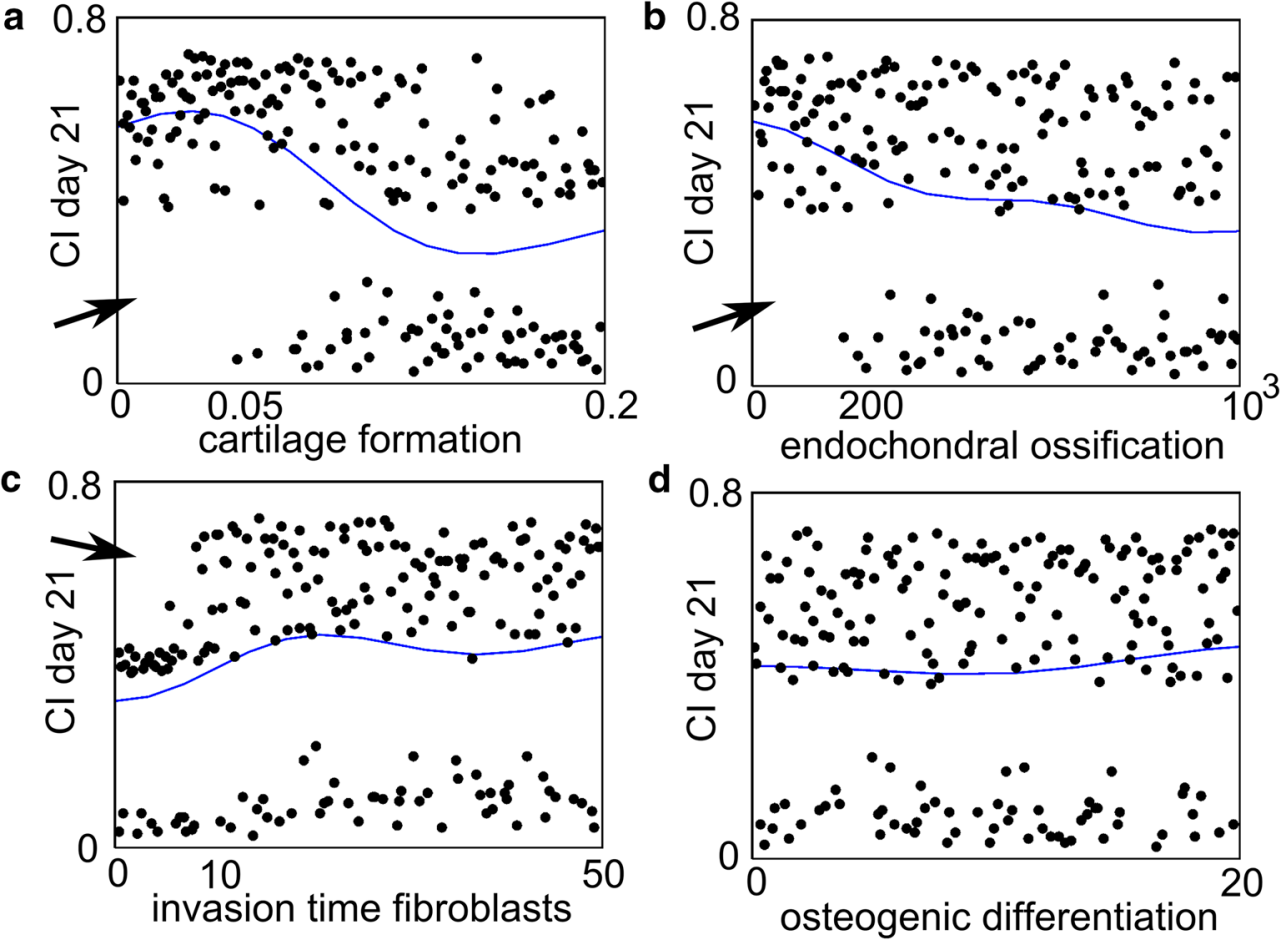
Results of the sensitivity analysis of the NF1 model. The horizontal axis shows the value of the parameter and the vertical axis shows the value of the complication index (CI) on Day 21. The CI value assess the degree of severity of CPT and is an averaged value of the amount of fibrous tissue, fibroblasts and bony union. A parameter combination for which the value of CI is small, is one for which the degree of severity of CPT is small (the fracture healing proceeds fairly normally). The dots indicate the actual results obtained from the computational model (each *dot* represents the CI response obtained for a particular combination of parameter values of the NF1 model), and the solid line indicates the statistical prediction by a Gaussian process. Note that the actual results (the *dots*) split up in two classes: one for which the CI value is high, resulting in impaired bone healing and one for which the CI value is low. In case (**d**), the dots are present in both classes, regardless of the parameter values. In cases **a**, **b**, parameter values below 0.05 and 200 respectively, always lead to a high CI value (indicated by the *arrow*), whereas in case (**c**) parameter values below 10 result in low to intermediate CI values (indicated by the *arrow*) (adapted from [50], licensed under CC BY 4.0)

Congenital pseudarthrosis of the tibia (CPT) is an orphan disease with an incidence of 1 per 53 000 births [58]. The clinical presentation varies between a primary pseudarthrosis at birth to (extensive) anterolateral bowing of the tibia during early infancy and spontaneous fractures in the distal third of the tibia when the child begins walking [19, 59]. Although the exact etiology of CPT is highly debated, 40–80 % of the CPT-patients are carriers of a mutation in the *Neurofibromatosis type 1* (*NF1*) gene, which can potentially result in an altered phenotype of the skeletal cells and impaired bone healing. To examine the effect of the *NF1* mutation on bone fracture healing, Carlier et al. altered the parameter values of the factors describing the aberrant cellular behaviour of *NF1* haploinsufficient and *NF1* bi-allelically inactivated cells in an established computational model of bone fracture healing [50]. The computational results showed that a combination of aberrant processes in skeletal cells, attributed in literature to the presence of a NF1 mutation, may lead to the prediction of a non-union including large quantity of fibrous tissue and limited endochondral ossification [50]. The relative importance of the eight altered factors to the model outcome was further explored in a large sensitivity analysis. Interestingly, the results of the sensitivity analysis clustered in two classes, one corresponding to impaired healing and one to normal healing (Fig. 6). A closer look at these findings indicated that the rate of cartilage formation (Fig. 6a), the rate of endochondral ossification (Fig. 6b) and the duration of fibroblast invasion (Fig. 6c) were the most important determinants of this behavior. Consequently, the computational model suggests that future research efforts should be focused on the characterization of the endochondral ossification pathway in *NF1* haploinsufficient and *NF1* bi-allelically inactivated cells as well as that of the invasion of lesional cells in the fracture callus in order to unravel the exact etiology of CPT and improve current treatment strategies.

Clearly, this type of approach can give interesting insights into the varieties in phenotypes that can result from defects in a single gene. Even though at this moment it is not feasible to link patient-specific characteristics to a particular combination of model parameters, valuable information can be obtained at the population level. Currently we are testing treatments involving BMP administration in the entire simulated population in order to understand the cause of the highly variable outcome of this treatment reported in the literature [60–62]. As such, this case-study represents an important step towards real in silico clinical trials, where it is envisioned that computer models would be able to predict the efficacy of a “virtual” treatment on a group of “virtual” patients [63]. Such in silico clinical trials could help to refine and reduce the size and duration of real clinical trials through better a design. Moreover, they have the potential to complement and replace real clinical trials, which would be particularly promising for orphan diseases (such as NF1-associated CPT), where only small cohorts of patients are available for clinical investigation, and for pediatric diseases where clinical trials are nearly impossible for ethical reasons.

### Non-union due to mechanical overload

**Fig. 7:**
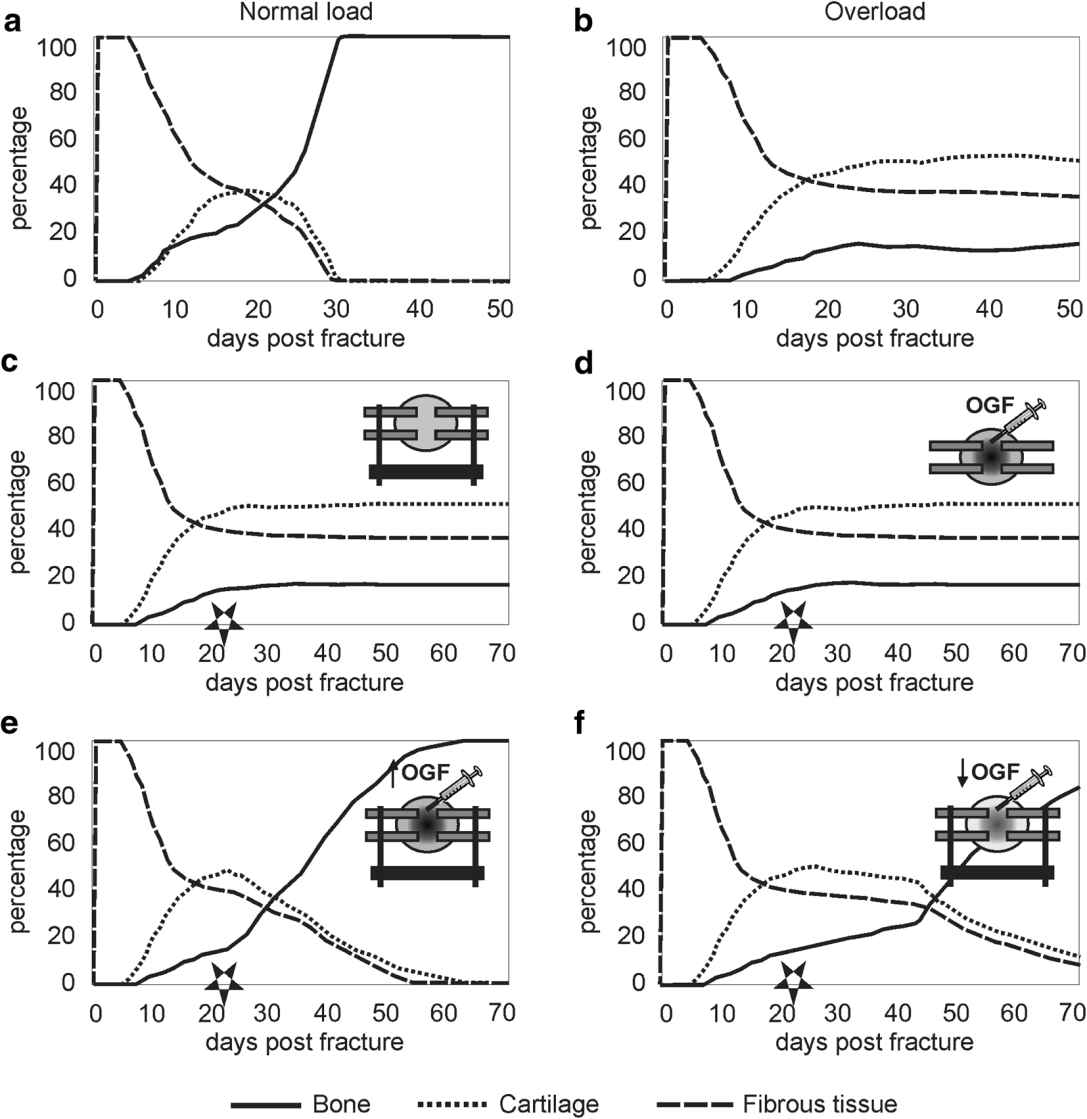
Fracture healing evolution under several loading conditions and treatment strategies. Evolution of the fibrous, cartilaginous and bone tissue fractions in the fracture callus under several loading conditions (the mechanical stimulus (i.e. interstitial fluid flow) influences angiogenesis, intramembranous and endochondral ossification): **a** healing under normal biological and mechanical conditions, **b** overloading (400 % of the normal load). **c**–**f** depict the results of various treatment strategies for overload-induced non-unions (the mechanical stimulus (i.e. interstitial fluid flow) influences proliferation, osteogenic differentiation, bone matrix production and endochondral ossification). Treatments of overload-induced non-unions started at 3 weeks post fracture (PFW3) as, by then, the first signs of endochondral ossification should have been visible in a normal healing process. The *star symbol* indicates the timing of the schematically depicted treatment. **c** stabilization of the callus area after 3 weeks of healing under unstable conditions (overloading) does not result in recapitulation of normal-healing processes, **d** administration of additional osteogenic growth factors (OGF, g_b_) at PFW3 without stabilization does not result in recapitulation of the normal healing process, **e** stabilization of the callus area in combination with the administration of sufficient osteogenic growth factors (0.1 mg/ml1) at PFW3 results in recapitulation of the normal healing process. **f** stabilization of the callus area in combination with the administration of osteogenic growth factors in a lower concentration (0.01 mg/ml) at PFW3 results in a slower recapitulation of the normal healing process when compared with (biv). (with kind permission from Springer Science + Business Media: Geris et al. [51], Fig. 6; adapted from [51, 64])

As stated above, adverse mechanical loading is known to be a major risk factor of delayed unions and (hypertrophic) non-unions [7]. However, the exact mechanisms by which mechanical (over)loading influences the regenerative processes during fracture healing are unknown. Geris et al. used a mechanobioregulatory model to investigate the influence of local mechanical stimuli on the angiogenic and osteogenic processes resulting in non-unions [51]. To simulate the effects of mechanical loading, interstitial fluid flow and hydrostatic pressure were chosen as mechanical stimuli, influencing either angiogenic parameters (i.e. endothelial cell proliferation, blood vessel synthesis, VEGF production by chondrocytes, blood vessel degradation) or a combination of parameters related to angiogenesis and intramembranous (i.e. osteoblast proliferation, MSC differentiation to osteoblasts, bone synthesis) and endochondral ossification (i.e. chondrocyte replacement) (Fig. 3). Solely in the case of a combined influence of mechanical loading (interstitial fluid flow) on angiogenesis and intramembranous and endochondral ossification, a non-union due to mechanical overloading was predicted (Fig. 7b). Further analysis of these simulation results showed that the required angiogenic factors are present in the fracture callus but that the adverse mechanical environment prevents the new vasculature from forming. If the local mechanical stimuli influence only angiogenesis, full bony bridging is observed for both normal and overloading conditions. For intermediate loading, the initial healing response was slightly delayed but this was compensated by a faster endochondral reaction (results not shown). In the case of underloading, an incomplete union (bridging without endochondral ossification in the periosteal callus) developed (results not shown). The above findings are corroborated by experimental and clinical studies emphasizing the importance of appropriate loading conditions for normal progression of bone regeneration. Interestingly, Geris. et al. [64] demonstrated that, depending on the dominating biology-mechanics interactions that are implemented in the computational model, different treatment strategies are required for the restoration of normal healing. For example, when proliferation, osteogenic differentiation, bone matrix production and endochondral ossification are the most influenced by mechanical loading, both adequate stabilization of the fracture environment and administration of sufficient osteogenic growth factors are necessary to result in complete healing (Fig. 7c–f). However, when me*c*hanical loading mainly influences proliferation, osteogenic differentiation and bone matrix production, the administration of osteogenic growth factors leads to a bony union 3 weeks after treatment, with or without removing the overload conditions (results not shown) [64]. From these results we can conclude that under comparable mechanical and biological conditions, the bone healing outcome can substantially differ from one patient to the other.

## Prospects

The above examples clearly illustrate that in silico techniques are able to investigate the etiology of a wide range of fracture non-union types and design novel treatment strategies thereof. More specifically, the first case-study demonstrated that the computational model can capture the essential aspects of an in vivo atrophic non-union and can help to explain and optimize experimental treatments, i.e. the location of the injection of a cell transplant. Similarly, the predictions of the second case-study showed that the effectiveness of a therapy, consisting of a single injection of osteochondrogenic growth factors, cells or a combination thereof in a large segmental bone defect, is strongly influenced by the (patient-specific) host environment and by the timing of injection. Moreover, case-study four evidenced that, depending on the dominating biology-mechanics interactions that are implemented in the computational model, different treatment strategies are required for the restoration of normal healing. As such, the results of these three case-studies clearly underline the need for patient-dependent modeling. However, at this moment it is not (yet) feasible to link patient-specific characteristics to a particular combination of model parameters (an in depth discussion of the key challenges associated with patient-specific modeling can be found in [65]). Nevertheless, case-study three nicely shows that the current approach can give interesting insights at the population level and represents an important first step towards in silico clinical trials.

The current computational framework has some limitations and the interpretation of the results should therefore be done carefully due to the following reasons. Firstly, the computational model only includes the repair phases of fracture healing, i.e. the soft and hard callus phase, and neglects the early inflammatory response and the bone remodeling phase. Other fracture healing models have accounted for the remodeling phase such as the work of Gómez-Benito et al. [66], Burke et al. [67], Byrne et al. [68] and Shefelbine et al. [69]. Moreover, in the literature also detailed models exist of the bone remodeling process itself, including the studies of Ryser et al. [70, 71] and Buenzli et al. [72–74]. However, none of the state-of-the-art fracture healing models, to the author’s best knowledge, captures the inflammatory phase.

Secondly, the presented computational framework requires a simplified and fixed geometrical domain of a fracture callus (Fig. 3). As such, the implementation cannot account for tissue growth during callus formation, although this can be captured in the frameworks of Chen et al. [75], Simon et al. [76] and Gomez-Benito et al. [66]. Moreover, the computational framework only allows 2D or 2D-axisymmetric calculations while others have simulated the regeneration processes in the inter-cortical region in 3D [68, 69, 77].

Thirdly, the computational model is based on experimental data from mouse models since these small animal models are increasingly used in bone healing studies due to their less expensive housing, shorter breeding cycles, well-defined genetic background and available (genetic) methods to study particular molecular mechanisms of action [78]. However, rodents have a more primitive bone structure without a Haversian system and use resorption cavities for bone remodeling, which is different from large animals and adults [78]. Moreover, in order to correctly mimic fracture healing in adults, animals of an age with completed bone growth should be used [79]. Given that the computational model is corroborated with experimental data from mouse models, it is important to keep in mind the differences that exist between murine and human bone healing when extrapolating these findings to a clinical setting. Note that others have used ovine models to explore the predictive power of computational models of bone healing. Moore et al. report for example that the histological measures (amongst others Giemsa-Eosin staining and fluorochrome microscopy) match the predicted gradients in BMP, cells and tissue fractions over time in an ovine critical size defect model [80]. Similarly, Chen et al. corroborate their predictions on the induction of non-unions in large gap sized and different mechanical conditions with experimental results obtained from an osteotomized ovine metatarsus [75].

Despite the above mentioned advances, several steps need to be taken in order to bring in silico models from bench to bed side [65]. These steps include, amongst others, the establishment of patient-specific models as well as their corroboration in both small (e.g. mice) and large (e.g. sheep) animal models and a limited number of patient-specific study cases. Although this road to translation is challenging, we believe that it is important to focus future research efforts to overcome these challenges so that computational models of bone fracture healing are not only used as research tools in the experimental research phase but also aid in the advancement of individualized care and reduction of the associated health care costs.

## Conclusion

In case of injury, the majority of bone fractures can heal without the production of scar tissue. Unfortunately, 5–10 % of the bone fractures fails to heal and develops into a non-union. This review illustrated the potential of computational models of fracture healing in contributing to a more profound understanding of the etiology and treatment of fracture non-unions. Four different cases of non-unions were discussed: non-union induced by periosteal and endosteal injury, non-union due to a large interfragmentary gap, non-union due to a genetic disorder (i.e. NF1 related congenital pseudoarthrosis of the tibia (CPT)) and non-union due to mechanical overload. Clearly, a treatment will be most beneficial if it tackles the underlying mechanism of action causing the hampered bone formation. The underlying mechanisms of action are, however, the result of complex non-linear biological and mechanical interactions occurring at various temporal and spatial scales. As such, a rigorous approach where in vivo and in silico methods work in tandem, are essential to deepen our fundamental understanding of (impaired) bone regeneration, to corroborate the existing computational models and to bring novel treatment strategies for challenging orthopedic cases from bench to bed side.
